# Life and Character of Chapin A. Harris, M. D., D. D. S.

**Published:** 1861-02

**Authors:** James Taylor


### LIFE AND CHARACTER OF CHAPIN A. HARRIS, M. D., D. D. S.

AN ADDRESS,

BY PROF. JAMES TAYLOR.

[Read before the Cincinnati Dental Association.]

Mr. President and Gentlemen:—In accordance with your kind and courteous request, I now endeavor to bring before you, in review, something of the life and character of one whose memory is dear to us all. You all know that Professor C. A. Harris is dead—that Death has indeed passed before us with solemn tread, and, with fatal shaft, has struck down the author, the friend of dental science, the friend of all who love dental progress, the pioneer of medico-dental education, and the zealous and indefatigable defender of the claims of dental surgery.

The solemn notes announce his demise—they tell us he is no more—and we ask, shall we no more hear his friendly greeting, no more grasp that hand so cordially extended, no more see that beaming countenance, radiant with the reflections of a pure and generous heart, no more hear that voice, ever ready with kind word and persuasive eloquence to cheer the youthful aspirant for professional excellence? That pen, so often wielded for the elevation of our science, lies silent and still, no more to trace the thoughts which welled up from the depths of his vast intellect, rich with stores of knowledge from every department of science, all contributory to that specialty he had chosen as his pursuit.

Some men, indeed, do die. They pass away, and are soon forgotten. But it is not so with Dr. Harris; for, though all these moving realities of life with him have passed away from us, and we shall see them no more, yet our friend still lives. He lives, fresh in our memories. He lives in our offices. We go into our laboratories, and he is there. We open our book cases, and it is Dr. Harris, *Dr. Harris*, DR. HARRIS. We can almost hear his voice, and see his radiant smile. That pen, though still now, traced thoughts that will never die. Dr. Harris "is not dead, but sleepeth," and will come forth at the great day to render up an account of his stewardship. Such a man we are proud to call our brother; and we feel that the lives of such men have rendered our profession an essential and invaluable service. May we all be able to do as well for our race!

We have met, my friends, this night to look at some of the excellences of the life and character of our departed brother. I feel that we can not fail to be bettered by the retrospect through which I shall endeavor to lead you. It may, indeed, urge on some of you that are now young, and just entering on the duties of the profession he so much adorned, to a like consecration of your time and talents.

PROF. CHAPIN A. HARRIS was born in Pomfrey, Onondaga county, New York, May 6th, 1806. He died, September 29th, 1860. His ancestors were originally from England; and their connection traces back to the author of "Hermes," James Harris, grandfather, I believe, to the present Earl of Malmsbury. Captain Joshua Harris, who fought bravely under Stark, at Bunker Hill, was his grand-uncle; and his grandfather was killed in a skirmish, during the Revolutionary

War. But Dr. Harris wrought out his own patent of nobility —one conferring more real honor than that derived from titled inheritance, because honestly earned, and always honorably maintained.

At about the age of seventeen, Dr. Harris came to Madison, Ohio, to which place his elder brothers, James and John, had previously removed. Here his brother John was practicing medicine, and he entered his office as a student. After pursuing the course of study which was then prescribed by the laws of the State, he applied to the Medical Censors, and was licensed to practice Medicine and Surgery. This State was then divided into quite a number of districts, in each of which a Board of Medical Censors, or Examiners, was appointed, whose duty it was to examine all candidates who applied for license to practice medicine; and without a certificate, or diploma, from this Board, no fees for medical services could be collected. This law appeared to be necessary to meet the want of proper medical institutions, which, up to this time, had done but little for medical education in the West. I believe that a diploma from a medical school rendered a certificate from a board of censors unnecessary; but, at that time, not more than one in ten of our regular physicians had a diploma from a medical college. The medical department of Transylvania University, at Lexington, Ky., and the Ohio Medical College were then just struggling into existence. It appears that the law above referred to did not include dental surgery; for dentists were not obliged to meet its requirements. Any one who saw fit, could cut, file, and tear away at the teeth without fear of law; and many, I fear, did so without much regard to gospel.

Dr. C. A. Harris, having received his license from the board of censors, commenced the practice of medicine in Greenfield, Ohio; and here my personal acquaintance with him began. His brother, Dr. John Harris, had, by this time, removed from Madison to Bainbridge, Ohio, my native place; and I was a student of medicine in his office. Dr. John Harris must have located in Bainbridge in 1826, and Dr. C. A. Harris, in Greenfield, in 1827. In this latter year, the former turned his attention to the practice of Dental Surgery. He had acquired considerable reputation as a general surgeon, and was a skillful and ready operator. He was much devoted to the study of anatomy and chemistry, fond of experimenting, had a quick and active mind, was polished in his manners, delighted in imparting instruction to his pupils, devoted much time to their interests, and prided much in their advancement in medical knowledge. At that time he had three or four students, two of whom followed his example in the pursuit of dental knowledge, myself being one of them. Being among my relatives and friends, and acquainted in all the surrounding towns, I had fine opportunities for putting into practice the little knowledge acquired.

The insertion of artificial teeth being then something of a novelty, Dr. John Harris and myself extended our practice to some of the adjacent towns, and, while thus engaged, visited Greenfield in 1828. Dr. C. A. Harris being there at the time, we operated, to some extent, in his office, and among his patrons and friends, and here began, on his part, that career of dental study, which expanded and strengthened, till it culminated in a rich stock of dental knowledge, for the good of the profession. He remained in Greenfield only about one year, and then removed to Bloomfield, Ohio, combining the practice of dentistry with his other duties, as occasion offered, for two or three years.*

We next find him wending his way eastward into Virginia, and having arrived at Fredericksburg, he began the practice of dentistry there, and his success was such that he abandoned entirely the practice of general medicine and surgery. But, though abandoning the practice, he by no means neglec-

* In an obituary notice I prepared for the Dental *Lamp*, I find that I erred in stating that Dr. Harris removed from Bloomfield to Greenfield. The reverse appears to have been the case. For some facts in the life of Prof. C. A. Harris, I am indebted to the kindness of his son, Mr. C. B. Harris, now of Baltimore.

ted the study of medicine; for his attainments were such, in this line, that more than once he received the honorary title of "Doctor of Medicine." Owing to his good success, as a dentist, he was no longer content with a small field for operating, and accordingly, he left Fredericksburg and came to Baltimore, which had many advantages for the practice of dentistry. Here, it may be said, his great work for the profession began. Here his energies were concentrated, and here was accomplished that which so endeared him to his professional brethren.

Dr. Harris' early advantages were not better than those of the most of you now before me. He began life, if not poor, at least not rich; and he had not, and could not then have any special advantages in dental instruction. Dentistry was then principally in the hands of quacks and empirics. The medical profession had not really cast it off; for they had never put it on. It was not, however, claimed as a part of medical science. It was looked upon, by physicians, as merely a mechanic art and was still so rude as to be left almost entirely to the hands of silversmiths, if not to tinkers. They regarded it as derogatory to their high calling to descend from the curing of disease, to dealing with the dental organs, further than to tear them from their sockets when their presence could no longer be endured.

Dr. John Harris and myself had many protracted discussions on the importance of a thorough medico-dental education, and the best method of securing it; and in these we were joined by Dr. C. A. Harris, on one or two occasions, or perhaps oftener. The leading idea, for several years, was to have a department of dental surgery attached to medical colleges. But the medical faculties had already too much to teach; and it was feared that while, by this course, all might be made dabblers in dental practice, but few would be made proficient in dental science. The more our specialty was looked at, the more important it appeared, and it soon became too large for annexation in that way. Prof. C. A. Harris certainly is entitled to the credit of making the first movement in the right direction, and of establishing that system of instruction which alone can give character and stability to our profession. I am truly glad to see that this system gains more and more in favor, and I can not see why any member of the profession should hold back, in regard to that which exalts and dignifies his own profession and himself.

It appears that in the early establishment of the Baltimore school, Dr. Harris met with some of that opposition which such enterprises are generally doomed to encounter. Many of the profession either opposed, or rendered no aid; and the medical department of the University of Maryland opposed the measure. There always appears to be interests, or monopolies, which are afraid of shadows, or over-jealous of their rights. I well remember when there was as much indifference, by medieal men, to the establishment of medical schools, as there is now, or rather as there was ten years since in relation to dental institutions, on the part of dentists. It takes time to wear off these fossil attachments. The march of science will and must overcome all such obstacles. The mighty power of truth will ultimately prevail and triumph.

How seldom is it that the originators of great enterprises live to see the entire success of their schemes! Dr. Harris, however, lived to see much of his labor crowned with success, but not long enough to derive that full amount of pecuniary profit which his arduous labors deserved.

Let us take a hasty glance at some of his professional labors, after his removal to Baltimore.

In 1839, we find him engaged in organizing the Baltimore College of Dental Surgery, which went into operation in the fall of that year. We find the following notice, which we believe is the first published by the American Journal of Dental Science, in relation to this school, and which, although written before Dr. Harris had any control of its pages, expressed his sentiments:

"There is no good reason (the Editor remarks) for the inferior rank that dental surgeons have been compelled to take in comparison with professional men. Why is the surgeon, who amputates a limb, or dresses an ulcer, more highly to be esteemed than he who confines his attention to the diseases of the mouth and dental arch? Is the knowledge of the former acquired with more laborious industry? Is his skill the result of more persevering research and careful experience? Do the operations he is called upon to perform require more judgment, or greater nicety than those which devolve upon the practitioner of dentistry? No man acquainted with the facts in each case, will respond affirmatively to these inquiries.

He who devotes his life to the alleviation of the suffering of his fellows, is a *respectable* man; and he who brings knowledge, carefully acquired, to aid in this object, is a *scientific* man. It matters not whether his efforts be directed to assuage a fever or remove a pain, to save life or to render it comfortable. The object, the honor, the motive is the same, and so it should be, in the regard of those who are profited thereby."

We have here the true secret of that continuous perseverance which impelled the Doctor to labor in this department of the healing art. Nobly casting aside all those prejudices which had been brought to bear on the practice of dentistry, he went zealously to work to redeem our science from obloquy, and knew no faltering until death removed him from his labors. It requires no little labor and self-denial to assume the responsibilities of a teacher in a school already organized and under successful operation; but not a tithe of that labor and anxiety which Dr. Harris must have undergone, in developing and perfecting a new enterprise, the educational materials of which had to be culled from a mass of scientific matter, really more voluminous than that which enshrines medical practice in general; for it branches out and takes in mineralogy, metallurgy, and mechanism.

Having engaged in this same educational enterprise so soon after my worthy colleague, I can speak with some knowledge on this matter; and hence, I can unhesitatingly say that the labor of preparing a course of lectures on any specialty of general medicine, so called, having regular text books for a standard, would not half equal that which is necessary in cutting out a new course without any such aid.

But all this, perhaps, does not equal that amount of labor, care and anxiety which is needed in founding and putting into active operation schools of this kind.

Look, gentlemen at the programme of study now and when our dental colleges were first organized. Truly we are now no more fit to be *annexed to*, than were our medical colleges fifteen and twenty years ago. We, like them, then, have as much as we can teach.

Time will not permit me to dwell on what must have been Dr. Harris' experience and toil, in the establishment of the Baltimore College of Dental Surgery. I have merely alluded to it in this place, feeling that I can appreciate it full as well as any man living.

We next find Dr. Harris engaged as one of the editors of the American Journal of Dental Science. His editorial duties began with the second volume of this valuable publication; and here, I have no doubt, was commenced that career of authorship which has done full as much as anything else to impress his labors on the profession for ages to come. Here, again, these new duties must have taxed to their utmost all those energies not already employed. Perhaps no man knows how to economize all his time, and exert all his talents, until forced from necessity to make the most of them. Up to 1850, other gentlemen assisted in these duties; yet without any disparagement to any of them, it must be apparent that the chief duty fell upon Dr. Harris. In 1850, he assumed the entire editorial control of the journal; but, from time to time, he has since been assisted by different members of the dental and medical professions, and was no doubt thus relieved of much arduous labor. Yet we find that, for some eighteen years, he held on to his post, his energy suffering no abatement, and his zeal never flagging.

Dr. Harris has been a voluminous author. His first work, which drew special attention, was his "Principles and Practice of Dental Surgery." This rapidly passed through six editions, and it is needless to say that it has, so far as the original scope of the work would allow, kept pace with the rapid improvement in the profession.

"Harris' Dictionary of Dental Science, Biography, Bibliography and Medical Terminology," published first in 1849, has passed through its second edition. We have still a later work—"Fox and Harris"—a reprint of an excellent old English work, with notes, &c., by Harris. These publications all show the careful and labored research of Prof. Harris; and the profession can not well over estimate the advantages derived from them.

Time would not permit a careful analysis of all these works, as well as the many essays and practical articles for our journals, from the pen of our author. We all have access to these works, and they speak for themselves. I therefore leave them where they will always remain, in the hands of the profession, and take a more close survey of his life and character—that which constitutes and makes up the man.

First.—He was a laborious student. This laid the foundation of all his usefulness and fame. His mind was not brilliant, but it was stable and comprehensive Prof. Harris, from the very moment that he determined on Dental Surgery as a vocation, became a zealous and indefatigable student, grasping everything which could be made to contribute to the advancement of dental science. Fortunately, before his thoughts were turned in this direction, he had studied medicine, and hence his mind at once saw the need of all the medical knowledge he could acquire in the practice of this, his chosen specialty.

We have every reason to believe that seeing the need of more cultivation in this department impelled him, as much as any thing else, to engage in its pursuit. We do know he felt that a very important branch of medical science was almost entirely neglected by the Medical Faculty, and that it was some one's duty to attend to it. Here was a neglected field, uncared for by the profession. A desert waste, overrun with weeds, foul and rank, choking out all the good seed, which had been here and there dropped by the way. To cultivate this field, to uproot these weeds, and make dental science a flourishing plant of the parent stem, would require much labor and assiduous toil. He willed it should be done, and, with no wavering spirit, he set about the work. His perseverance knew no weariness, his zeal no discouragement. This desert waste was to be fortified and made productive. It was to blossom out full of beauty, and rich with the choicest treasures of science. To accomplish this, Dr. Harris devoted all his latent energy and life.

Unfortunately, too many in all our professions feel that when the duties of practice are assumed they are *prepared*, and have but little more to learn. Dr. Harris did not so view this matter. He felt that perfection is rarely, if ever, attained in any pursuit; and saw, as difficulty after difficulty was overcome, in the manipulation of our art, more and more excellence in prospective. We have no doubt at all that, until disease had so laid her heavy hand upon him as to force out all thoughts of everything professional, did he ever give over his zeal in the pursuit of that perfection in dental science which he felt was so essential in practice. He almost idolized his profession, and made every thing in science succumb to her demands. He ransacked medical science in every department, for contributions to dentistry. He sifted the whole arcana of dental and medical literature and gleaned therefrom all the grain of very much value, tested all this by his own practical experience, improved, modified and developed, until he condensed in one work, more practical matter than can be found in any other one volume of the profession.

Talk of two, three or four years' studentship. Here was a labor of years—a studentship of over thirty years. May it not be that more real knowledge of the profession was obtained in the last year of his life than any other? As the mind by study enlarges and expands, it is better able to distinguish the grain from the chaff, and thus lay up a more precious and valuable stock of knowledge each successive year. Every year less and less chaff has to be analyzed. Thus, and in this way alone, can study become a pleasure.

The second prominent trait in the character of Dr. Harris was his fixedness of purpose—his stability. He did not commence a work, and throw it aside for every trifle; but he went straight on to the accomplishment of the object in view, making first this, and then that, stand aside out of his way, and bringing all the energies of his mind to bear on whatever he undertook.

No fickle, unstable man could have accomplished half which he did. Look at his labors as an author, to which I have referred, his labors for the college, his labors for the journal, his labors for our societies; and then add to all this an extensive and laborious practice, and you can form some conception of that fixed, indomitable perseverance, which must have reigned paramount in his character.

We find him at his post in the college, for twenty years—ever learning and ever ready to teach others what he had learned. He laid out his work carefully, looked over the whole field of labor, and then diligently and persistently went to work, waiting, year after year, for those results which he knew must come. Rest assured, young gentlemen, this is as sure as cause and effect. Let me urge upon you who are now just embarking in the same profession, to thus lay out your plans, economize your time, systematize all your labors, and never lay aside your studies,—do all this, and you need never trouble yourselves about *success*.

It is a noble sight to see talent thus dedicated to a good work; and while Dr. Harris thus dedicated himself to the advancement of dental science, and accomplished so much in this way, I have every reason to suppose that he neglected not those more important interests which lay hold on eternity.

As a father, a friend, and a good citizen, he will be sadly missed, and long truly mourned. It would be, I have no doubt, profitable to us all, to trace out this phase of his life; but time will not permit, and it more specially devolves on those recently more identified with him.

Dr. Harris' life very forcibly illustrates what can be accomplished by a fixed purpose, to overcome those obstacles which dishearten and paralyze the efforts of ninety-nine in every hundred of those engaging in scientific pursuits. To such, obstacles become the mere ordinary rebuff of every day's experience, and are overcome, as mere pigmies thrown in the way, to excite to renewed labor and zeal. Such minds expand and develop under difficulties. They expect not to glide smoothly down life's current, and be constantly fanned by gentle zephyrs. It takes the storms of life to rouse the dormant energies and bring into active exercise the reserved power of the system.

Many of the greatest achievements of science have been accomplished under the most adverse circumstances. Science always favors the assiduous wooer. It is true devotion that must gather laurels to deck her temple.

Our friend thus wooed. Thus he gathered laurels for the temple of our science, and

"Having won
The bound of man's appointed years, at last,
Life's blessings all enjoyed, life's labors done,
Serenely to his final rest has passed;
While the soft memory of his virtues, yet,
Lingers like twilight hues, when the bright sun is set."

"Cheerful he gave his being up, and went
To share the holy rest that waits a life well spent."

